# (*E*)-Methyl 2-[(2*S*,3*S*,12b*R*)-3-ethyl-8-meth­oxy-1,2,3,4,6,7,12,12b-octa­hydro­indolo[2,3-*a*]quinolizin-2-yl]-3-methoxy­acrylate ethanol solvate

**DOI:** 10.1107/S1600536809017309

**Published:** 2009-05-29

**Authors:** Paulo Carvalho, Edward B. Furr III, Christopher McCurdy

**Affiliations:** a Department of Medicinal Chemistry, University of Mississippi, 417 Faser Hall, University, MS 38677, USA; b Department of Medicinal Chemistry and Department of Pharmacology, University of Mississippi, 417 Faser Hall, University, MS 38677, USA

## Abstract

In the title compound, C_23_H_30_N_2_O_4_·C_2_H_6_O, the indole derivative has four fused rings, forming an indolo[2-3*a*]quinolizine system, in which one six-membered ring is directly connected to the indole unit and has a distorted chair conformation. The fourth ring is also a six-membered ring, depicting a regular chair conformation.  In the crystal, the mol­ecules are linked by N—H⋯O and O—H⋯N inter­actions, forming a *C*(7) chain.

## Related literature

For previous crystallographic analysis of mitragynine salts (hydro­bromide and hydro­iodide), see: Zacharias *et al.* (1965 ▶). For the method of extraction, see: Ponglux *et al.* (1994 ▶). For synthetic studies, see: Ma *et al.* (2009 ▶). For medicinal properties, see: Boyer *et al.* (2008 ▶); Weibrecht *et al.* (2008 ▶). For hydrogen-bond motifs, see: Bernstein *et al.* (1995 ▶).
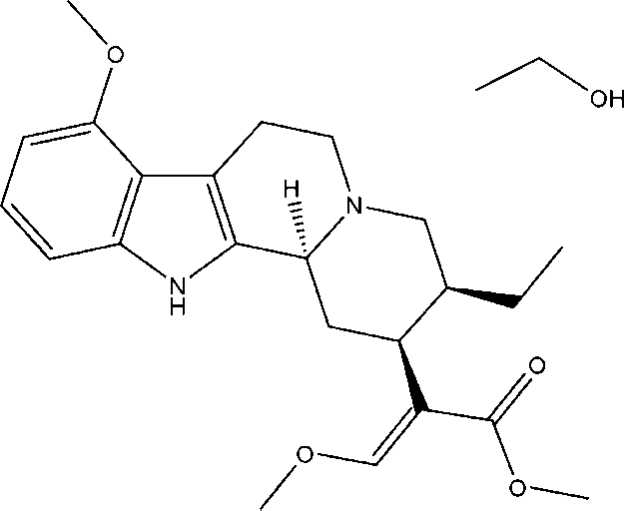


## Experimental

### 

#### Crystal data

C_23_H_30_N_2_O_4_·C_2_H_6_O
*M* *_r_* = 444.56
Orthorhombic, 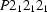

*a* = 7.60450 (10) Å
*b* = 11.7534 (2) Å
*c* = 26.5735 (4) Å
*V* = 2375.11 (6) Å^3^
*Z* = 4
Cu *K*α radiation
μ = 0.70 mm^−1^
*T* = 100 K
0.12 × 0.09 × 0.06 mm

#### Data collection

Bruker APEXII CCD diffractometer
Absorption correction: none
34365 measured reflections
4158 independent reflections
3649 reflections with *I* > 2σ(*I*)
*R*_int_ = 0.083

#### Refinement

*R*[*F* ^2^ > 2σ(*F* ^2^)] = 0.036
*wR*(*F* ^2^) = 0.089
*S* = 1.05
4158 reflections
295 parameters
H-atom parameters constrained
Δρ_max_ = 0.19 e Å^−3^
Δρ_min_ = −0.18 e Å^−3^
Absolute structure: Flack (1983 ▶), 1758 Friedel pairs
Flack parameter: 0.2 (2)

### 

Data collection: *APEX2* (Bruker, 2005 ▶); cell refinement: *SAINT* (Bruker, 2005 ▶); data reduction: *SAINT*; program(s) used to solve structure: *SHELXS97* (Sheldrick, 2008 ▶); program(s) used to refine structure: *SHELXL97* (Sheldrick, 2008 ▶); molecular graphics: *SHELXTL* (Sheldrick, 2008 ▶); software used to prepare material for publication: *SHELXTL* and *ORTEP-3* (Farrugia, 1997 ▶).

## Supplementary Material

Crystal structure: contains datablocks I, global. DOI: 10.1107/S1600536809017309/bx2206sup1.cif

Structure factors: contains datablocks I. DOI: 10.1107/S1600536809017309/bx2206Isup2.hkl

Additional supplementary materials:  crystallographic information; 3D view; checkCIF report

## Figures and Tables

**Table 1:** Hydrogen-bond geometry (Å, °)

*D*—H⋯*A*	*D*—H	H⋯*A*	*D*⋯*A*	*D*—H⋯*A*
O5—H5⋯N2	0.82	2.07	2.876 (2)	169
N1—H1⋯O5^i^	0.86	2.01	2.866 (2)	170

Symmetry code: (i) 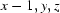.

**Fig. 1.**
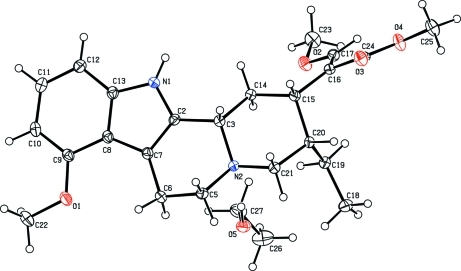
Molecular structure of the title compound, with the atom-numbering scheme. Displacement ellipsoids are drawn at the 50% probability level.

**Fig. 2.**
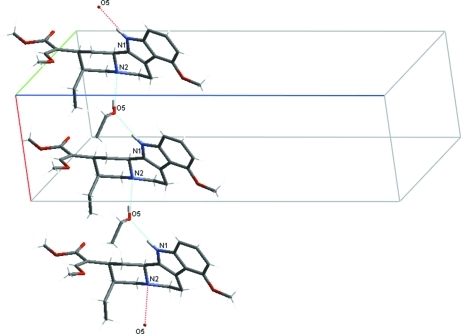
Part of the crystal structure of the title compound, showing the formation of a C(7) chain, along [100]. H atoms not involved in this motiv were omitted for the sake of clarity.

## supplementary crystallographic information

### Comment

Kratom (*Mitragynia speciosa* korth) is a medicinal herb endogenous to southeast Asia traditionally used as a treatment for opium withdrawal. Patients with chronic pain are increasingly aware of kratom as opioid replacement therapy. Mitragynine, the predominant alkaloid of kratom, binds with high affinity at human adrenergic, serotinergic, and adenosine *CNS* receptors. The binding affinity of mitragynine at mu (KD = 204 plus or minus 26 nM), delta (KD = 2250 plus or minus 47nM) and kappa (KD = 455 plus or minus 47 nM) receptors suggest that the mu-opioid agonism of mitragynine may minimize opioid withdrawal symptoms; as a kappa agonist, the molecule may oppose mu-opioid effects to modulatere inforcement and produce aversion. Furthermore, adrenergic agonist activity at alpha-2 receptors may permit kratom to mimic adjunctive therapies for opioid withdrawal such as clonidine (Weibrecht *et al.*, 2008). The title compound has four fused rings, forming an indolo[2-3a]quinolizine system, in which one six-membered ring is directly connected to the indol moiety and has a distorted chair conformation. The fourth ring is also a six-membered ring, depicting a regular chair conformation. The molecules are linked by N—H···O and O—H···N interactions, forming a chain C(7) (Bernstein *et al.*, 1995) along [100] directions (Fig. 2).

### Experimental

Mitragynine was extracted from dried *M. speciosa* leaves according to the procedure published by Ponglux *et al.*, (1994), and crystallized from a solution in ethanol.

### Refinement

All H atoms were located in difference maps and treated as riding atoms, with the following distance restraints: C—H = 0.93 Å, *U*_iso_(H) = 1.2*U*_eq_(C) for C*sp*2, C—H = 0.98 Å, *U*_iso_(H) = 1.2*U*_eq_(C) for CH, C—H = 0.97 Å, *U*_iso_(H) = 1.2*U*_eq_(C) for CH_2_, C—H = 0.96 Å, *U*_iso_(H) = 1.5*U*_eq_(C) for CH_3_, N—H = 0.86 Å, *U*_iso_(H) = 1.2*U*_eq_(N), O—H = 0.82 Å, *U*_iso_(H) = 1.5*U*_eq_(O).

### Crystal data

**Table:**

C_23_H_30_N_2_O_4_·C_2_H_6_O	*F*(000) = 960
*M**_r_* = 444.56	*D*_x_ = 1.243 Mg m^−^^3^
Orthorhombic, *P*2_1_2_1_2_1_	Cu *K*α radiation, λ = 1.54178 Å
Hall symbol: P 2ac 2ab	Cell parameters from 3152 reflections
*a* = 7.6045 (1) Å	θ = 3.3–64.6°
*b* = 11.7534 (2) Å	µ = 0.70 mm^−^^1^
*c* = 26.5735 (4) Å	*T* = 100 K
*V* = 2375.11 (6) Å^3^	Needle, colourless
*Z* = 4	0.12 × 0.09 × 0.06 mm

### Data collection

**Table:**

Bruker APEXII CCD diffractometer	3649 reflections with *I* > 2σ(*I*)
Radiation source: Sealed Tube	*R*_int_ = 0.083
graphite	θ_max_ = 66.4°, θ_min_ = 3.3°
φ and ω scans	*h* = −9→9
34365 measured reflections	*k* = −13→13
4158 independent reflections	*l* = −31→31

### Refinement

**Table:**

Refinement on *F*^2^	Secondary atom site location: difference Fourier map
Least-squares matrix: full	Hydrogen site location: inferred from neighbouring sites
*R*[*F*^2^ > 2σ(*F*^2^)] = 0.036	H-atom parameters constrained
*wR*(*F*^2^) = 0.089	*w* = 1/[σ^2^(*F*_o_^2^) + (0.0469*P*)^2^ + 0.2771*P*] where *P* = (*F*_o_^2^ + 2*F*_c_^2^)/3
*S* = 1.05	(Δ/σ)_max_ = 0.001
4158 reflections	Δρ_max_ = 0.19 e Å^−^^3^
295 parameters	Δρ_min_ = −0.18 e Å^−^^3^
0 restraints	Absolute structure: Flack (1983), 1758 Friedel pairs
Primary atom site location: structure-invariant direct methods	Flack parameter: 0.2 (2)

### Special details

**Table:**

Geometry. All e.s.d.'s (except the e.s.d. in the dihedral angle between two l.s. planes) are estimated using the full covariance matrix. The cell e.s.d.'s are taken into account individually in the estimation of e.s.d.'s in distances, angles and torsion angles; correlations between e.s.d.'s in cell parameters are only used when they are defined by crystal symmetry. An approximate (isotropic) treatment of cell e.s.d.'s is used for estimating e.s.d.'s involving l.s. planes.
Refinement. Refinement of *F*^2^ against ALL reflections. The weighted *R*-factor *wR* and goodness of fit *S* are based on *F*^2^, conventional *R*-factors *R* are based on *F*, with *F* set to zero for negative *F*^2^. The threshold expression of *F*^2^ > σ(*F*^2^) is used only for calculating *R*-factors(gt) *etc*. and is not relevant to the choice of reflections for refinement. *R*-factors based on *F*^2^ are statistically about twice as large as those based on *F*, and *R*- factors based on ALL data will be even larger.

### Fractional atomic coordinates and isotropic or equivalent isotropic displacement parameters (Å^2^)

**Table:**

	*x*	*y*	*z*	*U*_iso_*/*U*_eq_	
C3	0.3530 (3)	0.08591 (16)	0.79611 (7)	0.0226 (4)	
H3	0.2920	0.0127	0.7987	0.027*	
C2	0.3100 (3)	0.13687 (16)	0.74583 (7)	0.0224 (4)	
C14	0.2990 (3)	0.15900 (16)	0.84110 (7)	0.0224 (4)	
H14A	0.1750	0.1778	0.8388	0.027*	
H14B	0.3657	0.2293	0.8412	0.027*	
C5	0.6061 (3)	0.00338 (18)	0.75273 (7)	0.0269 (5)	
H5A	0.5360	−0.0647	0.7479	0.032*	
H5B	0.7275	−0.0197	0.7574	0.032*	
C16	0.2800 (3)	0.14709 (17)	0.93877 (7)	0.0237 (4)	
C21	0.5794 (3)	−0.00691 (16)	0.84300 (7)	0.0245 (4)	
H21A	0.7032	−0.0267	0.8439	0.029*	
H21B	0.5124	−0.0769	0.8405	0.029*	
C20	0.5303 (3)	0.05433 (16)	0.89183 (7)	0.0228 (4)	
H20	0.5406	−0.0014	0.9191	0.027*	
C7	0.4161 (3)	0.13592 (16)	0.70469 (7)	0.0234 (4)	
C11	0.0631 (3)	0.29780 (17)	0.60685 (8)	0.0301 (5)	
H11	−0.0215	0.3322	0.5867	0.036*	
C17	0.2551 (3)	0.25695 (16)	0.94916 (7)	0.0257 (4)	
H17	0.2198	0.2764	0.9815	0.031*	
C8	0.3190 (3)	0.19029 (16)	0.66512 (7)	0.0231 (4)	
C10	0.2287 (3)	0.27195 (17)	0.58613 (7)	0.0281 (5)	
H10	0.2523	0.2904	0.5528	0.034*	
C6	0.5923 (3)	0.07864 (19)	0.70579 (8)	0.0286 (5)	
H6A	0.6847	0.1355	0.7063	0.034*	
H6B	0.6070	0.0325	0.6758	0.034*	
C15	0.3348 (3)	0.09212 (16)	0.88976 (7)	0.0226 (4)	
H15	0.2658	0.0220	0.8871	0.027*	
C9	0.3566 (3)	0.21944 (17)	0.61479 (7)	0.0246 (4)	
C13	0.1537 (3)	0.21960 (17)	0.68469 (7)	0.0241 (4)	
C12	0.0231 (3)	0.27357 (17)	0.65616 (7)	0.0281 (4)	
H12	−0.0856	0.2921	0.6699	0.034*	
C19	0.6535 (3)	0.15316 (18)	0.90462 (8)	0.0268 (4)	
H19A	0.6527	0.2068	0.8769	0.032*	
H19B	0.6081	0.1923	0.9340	0.032*	
C18	0.8419 (3)	0.1177 (2)	0.91491 (9)	0.0359 (5)	
H18A	0.8946	0.0905	0.8844	0.054*	
H18B	0.8432	0.0583	0.9397	0.054*	
H18C	0.9071	0.1819	0.9272	0.054*	
C24	0.2472 (3)	0.06448 (17)	0.98062 (7)	0.0245 (4)	
C22	0.5687 (3)	0.2261 (2)	0.54835 (8)	0.0362 (5)	
H22A	0.5535	0.3068	0.5450	0.054*	
H22B	0.4941	0.1875	0.5247	0.054*	
H22C	0.6892	0.2066	0.5418	0.054*	
C25	0.2001 (4)	0.0389 (2)	1.06740 (8)	0.0398 (6)	
H25A	0.2861	−0.0208	1.0688	0.060*	
H25B	0.0856	0.0065	1.0623	0.060*	
H25C	0.2013	0.0808	1.0984	0.060*	
C23	0.2173 (4)	0.45082 (18)	0.93323 (9)	0.0393 (6)	
H23A	0.2795	0.4710	0.9634	0.059*	
H23B	0.0936	0.4468	0.9402	0.059*	
H23C	0.2384	0.5073	0.9079	0.059*	
C27	0.7975 (3)	0.33626 (19)	0.79406 (9)	0.0404 (6)	
H27A	0.6948	0.3492	0.8149	0.048*	
H27B	0.7713	0.3627	0.7603	0.048*	
C26	0.9507 (4)	0.4008 (2)	0.81476 (12)	0.0505 (7)	
H26A	1.0519	0.3881	0.7939	0.076*	
H26B	0.9750	0.3752	0.8484	0.076*	
H26C	0.9235	0.4806	0.8153	0.076*	
N2	0.5446 (2)	0.06406 (13)	0.79818 (6)	0.0223 (4)	
N1	0.1505 (2)	0.18655 (14)	0.73471 (6)	0.0237 (4)	
H1	0.0642	0.1955	0.7552	0.028*	
O5	0.83716 (18)	0.21863 (12)	0.79292 (5)	0.0297 (3)	
H5	0.7462	0.1818	0.7960	0.045*	
O3	0.2266 (2)	−0.03588 (11)	0.97486 (5)	0.0323 (3)	
O2	0.2782 (2)	0.34155 (11)	0.91554 (5)	0.0303 (3)	
O1	0.52291 (19)	0.19211 (12)	0.59849 (5)	0.0310 (3)	
O4	0.2410 (2)	0.11472 (11)	1.02612 (5)	0.0343 (4)	

### Atomic displacement parameters (Å^2^)

**Table:**

	*U*^11^	*U*^22^	*U*^33^	*U*^12^	*U*^13^	*U*^23^
C3	0.0226 (10)	0.0251 (10)	0.0201 (9)	0.0001 (8)	0.0008 (9)	0.0000 (8)
C2	0.0197 (10)	0.0247 (10)	0.0226 (9)	−0.0004 (8)	−0.0025 (8)	−0.0016 (8)
C14	0.0200 (10)	0.0273 (10)	0.0200 (9)	0.0005 (8)	−0.0001 (8)	−0.0007 (8)
C5	0.0234 (11)	0.0319 (11)	0.0253 (10)	0.0065 (9)	−0.0002 (8)	−0.0038 (8)
C16	0.0182 (10)	0.0317 (10)	0.0213 (9)	−0.0008 (8)	0.0002 (8)	−0.0010 (8)
C21	0.0229 (10)	0.0281 (10)	0.0226 (10)	0.0029 (8)	−0.0003 (8)	0.0007 (8)
C20	0.0224 (11)	0.0282 (10)	0.0177 (9)	0.0038 (8)	−0.0005 (8)	0.0026 (8)
C7	0.0236 (10)	0.0263 (10)	0.0204 (9)	−0.0017 (8)	−0.0004 (8)	−0.0023 (8)
C11	0.0329 (12)	0.0302 (11)	0.0273 (10)	−0.0005 (9)	−0.0086 (9)	0.0016 (9)
C17	0.0236 (11)	0.0309 (11)	0.0227 (10)	−0.0034 (9)	0.0005 (9)	0.0013 (8)
C8	0.0258 (11)	0.0237 (9)	0.0197 (9)	−0.0041 (8)	−0.0009 (8)	−0.0026 (7)
C10	0.0363 (12)	0.0287 (10)	0.0193 (9)	−0.0048 (10)	−0.0024 (9)	0.0007 (8)
C6	0.0255 (11)	0.0397 (12)	0.0207 (10)	0.0034 (9)	0.0019 (9)	−0.0028 (9)
C15	0.0209 (10)	0.0257 (10)	0.0213 (10)	−0.0012 (8)	0.0009 (8)	−0.0010 (8)
C9	0.0272 (11)	0.0255 (10)	0.0212 (9)	−0.0043 (8)	0.0018 (9)	−0.0041 (8)
C13	0.0262 (11)	0.0243 (9)	0.0218 (9)	−0.0031 (8)	−0.0015 (8)	−0.0015 (8)
C12	0.0260 (11)	0.0293 (11)	0.0291 (10)	−0.0008 (9)	−0.0026 (9)	0.0009 (9)
C19	0.0235 (11)	0.0340 (11)	0.0229 (10)	0.0016 (9)	−0.0020 (9)	−0.0024 (8)
C18	0.0241 (12)	0.0469 (14)	0.0366 (12)	0.0017 (10)	−0.0020 (10)	−0.0038 (10)
C24	0.0181 (10)	0.0314 (11)	0.0240 (10)	0.0005 (9)	0.0029 (9)	−0.0051 (8)
C22	0.0450 (14)	0.0369 (12)	0.0265 (11)	−0.0045 (11)	0.0109 (10)	0.0016 (9)
C25	0.0561 (17)	0.0359 (12)	0.0275 (11)	−0.0033 (11)	0.0084 (11)	0.0037 (9)
C23	0.0493 (15)	0.0280 (11)	0.0406 (13)	0.0020 (11)	0.0050 (11)	0.0023 (9)
C27	0.0389 (14)	0.0370 (12)	0.0452 (13)	0.0090 (10)	0.0105 (12)	0.0091 (10)
C26	0.0404 (15)	0.0308 (13)	0.0804 (19)	0.0015 (11)	0.0045 (14)	−0.0067 (13)
N2	0.0185 (8)	0.0288 (9)	0.0196 (8)	0.0024 (7)	−0.0006 (7)	−0.0012 (7)
N1	0.0217 (9)	0.0294 (8)	0.0201 (8)	0.0026 (7)	0.0026 (7)	0.0002 (7)
O5	0.0237 (7)	0.0330 (7)	0.0326 (8)	0.0005 (6)	0.0038 (7)	0.0013 (6)
O3	0.0413 (9)	0.0279 (8)	0.0276 (7)	−0.0012 (7)	0.0041 (7)	0.0005 (6)
O2	0.0394 (9)	0.0246 (7)	0.0269 (7)	0.0017 (7)	0.0045 (6)	−0.0020 (5)
O1	0.0348 (9)	0.0368 (8)	0.0215 (7)	−0.0023 (7)	0.0047 (6)	0.0005 (6)
O4	0.0527 (10)	0.0285 (7)	0.0216 (7)	−0.0041 (7)	0.0050 (7)	0.0012 (6)

### Geometric parameters (Å, °)

**Table:**

C3—N2	1.481 (3)	C6—H6B	0.9700
C3—C2	1.500 (3)	C15—H15	0.9800
C3—C14	1.528 (2)	C9—O1	1.375 (2)
C3—H3	0.9800	C13—N1	1.385 (2)
C2—C7	1.359 (3)	C13—C12	1.401 (3)
C2—N1	1.378 (3)	C12—H12	0.9300
C14—C15	1.538 (2)	C19—C18	1.517 (3)
C14—H14A	0.9700	C19—H19A	0.9700
C14—H14B	0.9700	C19—H19B	0.9700
C5—N2	1.479 (2)	C18—H18A	0.9600
C5—C6	1.533 (3)	C18—H18B	0.9600
C5—H5A	0.9700	C18—H18C	0.9600
C5—H5B	0.9700	C24—O3	1.200 (2)
C16—C17	1.334 (3)	C24—O4	1.346 (2)
C16—C24	1.497 (3)	C22—O1	1.434 (2)
C16—C15	1.512 (3)	C22—H22A	0.9600
C21—N2	1.478 (2)	C22—H22B	0.9600
C21—C20	1.530 (3)	C22—H22C	0.9600
C21—H21A	0.9700	C25—O4	1.447 (2)
C21—H21B	0.9700	C25—H25A	0.9600
C20—C19	1.530 (3)	C25—H25B	0.9600
C20—C15	1.553 (3)	C25—H25C	0.9600
C20—H20	0.9800	C23—O2	1.444 (3)
C7—C8	1.435 (3)	C23—H23A	0.9600
C7—C6	1.500 (3)	C23—H23B	0.9600
C11—C12	1.375 (3)	C23—H23C	0.9600
C11—C10	1.408 (3)	C27—O5	1.415 (3)
C11—H11	0.9300	C27—C26	1.495 (4)
C17—O2	1.348 (2)	C27—H27A	0.9700
C17—H17	0.9300	C27—H27B	0.9700
C8—C13	1.403 (3)	C26—H26A	0.9600
C8—C9	1.410 (3)	C26—H26B	0.9600
C10—C9	1.381 (3)	C26—H26C	0.9600
C10—H10	0.9300	N1—H1	0.8600
C6—H6A	0.9700	O5—H5	0.8200
			
N2—C3—C2	108.46 (15)	O1—C9—C8	115.38 (17)
N2—C3—C14	109.44 (16)	C10—C9—C8	119.27 (18)
C2—C3—C14	114.45 (16)	N1—C13—C12	129.34 (18)
N2—C3—H3	108.1	N1—C13—C8	107.59 (17)
C2—C3—H3	108.1	C12—C13—C8	123.07 (18)
C14—C3—H3	108.1	C11—C12—C13	116.90 (19)
C7—C2—N1	110.71 (16)	C11—C12—H12	121.5
C7—C2—C3	125.74 (18)	C13—C12—H12	121.5
N1—C2—C3	123.47 (17)	C18—C19—C20	114.17 (18)
C3—C14—C15	108.83 (15)	C18—C19—H19A	108.7
C3—C14—H14A	109.9	C20—C19—H19A	108.7
C15—C14—H14A	109.9	C18—C19—H19B	108.7
C3—C14—H14B	109.9	C20—C19—H19B	108.7
C15—C14—H14B	109.9	H19A—C19—H19B	107.6
H14A—C14—H14B	108.3	C19—C18—H18A	109.5
N2—C5—C6	111.38 (16)	C19—C18—H18B	109.5
N2—C5—H5A	109.4	H18A—C18—H18B	109.5
C6—C5—H5A	109.4	C19—C18—H18C	109.5
N2—C5—H5B	109.4	H18A—C18—H18C	109.5
C6—C5—H5B	109.4	H18B—C18—H18C	109.5
H5A—C5—H5B	108.0	O3—C24—O4	122.76 (18)
C17—C16—C24	116.77 (17)	O3—C24—C16	124.37 (17)
C17—C16—C15	129.11 (18)	O4—C24—C16	112.86 (16)
C24—C16—C15	114.11 (16)	O1—C22—H22A	109.5
N2—C21—C20	111.97 (15)	O1—C22—H22B	109.5
N2—C21—H21A	109.2	H22A—C22—H22B	109.5
C20—C21—H21A	109.2	O1—C22—H22C	109.5
N2—C21—H21B	109.2	H22A—C22—H22C	109.5
C20—C21—H21B	109.2	H22B—C22—H22C	109.5
H21A—C21—H21B	107.9	O4—C25—H25A	109.5
C21—C20—C19	113.32 (17)	O4—C25—H25B	109.5
C21—C20—C15	109.77 (16)	H25A—C25—H25B	109.5
C19—C20—C15	112.13 (16)	O4—C25—H25C	109.5
C21—C20—H20	107.1	H25A—C25—H25C	109.5
C19—C20—H20	107.1	H25B—C25—H25C	109.5
C15—C20—H20	107.1	O2—C23—H23A	109.5
C2—C7—C8	106.27 (17)	O2—C23—H23B	109.5
C2—C7—C6	121.23 (18)	H23A—C23—H23B	109.5
C8—C7—C6	132.39 (18)	O2—C23—H23C	109.5
C12—C11—C10	121.73 (19)	H23A—C23—H23C	109.5
C12—C11—H11	119.1	H23B—C23—H23C	109.5
C10—C11—H11	119.1	O5—C27—C26	109.7 (2)
C16—C17—O2	123.94 (18)	O5—C27—H27A	109.7
C16—C17—H17	118.0	C26—C27—H27A	109.7
O2—C17—H17	118.0	O5—C27—H27B	109.7
C13—C8—C9	118.26 (18)	C26—C27—H27B	109.7
C13—C8—C7	107.40 (16)	H27A—C27—H27B	108.2
C9—C8—C7	134.32 (19)	C27—C26—H26A	109.5
C9—C10—C11	120.73 (18)	C27—C26—H26B	109.5
C9—C10—H10	119.6	H26A—C26—H26B	109.5
C11—C10—H10	119.6	C27—C26—H26C	109.5
C7—C6—C5	109.66 (16)	H26A—C26—H26C	109.5
C7—C6—H6A	109.7	H26B—C26—H26C	109.5
C5—C6—H6A	109.7	C21—N2—C5	109.23 (14)
C7—C6—H6B	109.7	C21—N2—C3	107.72 (15)
C5—C6—H6B	109.7	C5—N2—C3	111.39 (15)
H6A—C6—H6B	108.2	C2—N1—C13	108.02 (16)
C16—C15—C14	117.18 (16)	C2—N1—H1	126.0
C16—C15—C20	110.83 (16)	C13—N1—H1	126.0
C14—C15—C20	110.24 (15)	C27—O5—H5	109.5
C16—C15—H15	105.9	C17—O2—C23	113.49 (15)
C14—C15—H15	105.9	C9—O1—C22	116.81 (16)
C20—C15—H15	105.9	C24—O4—C25	114.73 (16)
O1—C9—C10	125.35 (17)		

### Hydrogen-bond geometry (Å, °)

**Table:**

*D*—H···*A*	*D*—H	H···*A*	*D*···*A*	*D*—H···*A*
O5—H5···N2	0.82	2.07	2.876 (2)	169
N1—H1···O5^i^	0.86	2.01	2.866 (2)	170

Symmetry codes: (i) *x*−1, *y*, *z*.

## References

[bb1] Bernstein, J., Davis, R. E., Shimoni, L. & Chang, N.-L. (1995). *Angew. Chem. Int. Ed. Engl.* **34**, 1555–1573.

[bb2] Boyer, E. W., Babu, K. M., Adkins, J. E., McCurdy, C. R. & Halpern, J. H. (2008). *Addiction*, **103**, 1048–1050. 10.1111/j.1360-0443.2008.02209.x PMC3670991 18482427

[bb3] Bruker (2005). *APEX2*, *SAINT* and *SADABS* Bruker AXS Inc., Madison, Wisconsin, USA.

[bb4] Farrugia, L. J. (1997). *J. Appl. Cryst.* **30**, 565.

[bb5] Flack, H. D. (1983). *Acta Cryst.* A**39**, 876–881.

[bb6] Ma, J., Yin, W., Zhou, H., Liao, X. & Cook, J. M. (2009). *J. Org. Chem.* **74**, 264–273. 10.1021/jo801839t PMC2654583 19046119

[bb7] Ponglux, D., Wongseripipatana, S., Takayama, H., Kikuchi, M., Kurihara, M., Kitajima, M., Aimi, N. & Sakai, S. (1994). *Planta Med.* **60**, 580–581. 10.1055/s-2006-959578 17236085

[bb8] Sheldrick, G. M. (2008). *Acta Cryst.* A**64**, 112–122. 10.1107/S0108767307043930 18156677

[bb9] Weibrecht, K. W., Courtney, J. M., Halpern, J., McCurdy, C. & Boyer, E. W. (2008). *Clin. Toxicol.* **46**, 395–399.

[bb10] Zacharias, D. E., Rosenstein, R. D. & Jeffrey, G. A. (1965). *Acta Cryst.* **18**, 1039–1043.

